# Pulmonary vascular remodeling and right heart failure in pulmonary hypertension: future role of positron emission tomography in decoding the enigma

**DOI:** 10.1186/2213-0802-1-16

**Published:** 2013-12-11

**Authors:** Hendrik J Harms, Mariëlle C van de Veerdonk, Adriaan A Lammertsma, Anton Vonk Noordegraaf, Harm Jan Bogaard

**Affiliations:** 1 Department of Pulmonary Medicine, VU University Medical Center, PO Box 7057, Amsterdam, MB 1007 The Netherlands; 2 Department of Radiology & Nuclear Medicine, VU University Medical Center, Amsterdam, The Netherlands

**Keywords:** Pulmonary hypertension, Right heart, Positron emission tomography, Glycolysis, Proliferation, Extracellular matrix, Oxygen uptake

## Abstract

Whereas the insights into the cellular and molecular mechanisms of pulmonary arterial hypertension (PAH) and associated right heart failure have increased in recent years, there is a lack of clinical tools to assess the pathobiological mechanisms in patients. Positron emission tomography (PET) provides an array of new possibilities to image and quantify relevant disease processes, including proliferation, angiogenesis, matrix remodeling, shifts in metabolism and neurohormonal signaling. Here we describe the first studies which were conducted to image pulmonary vascular remodeling and right heart failure *in vivo* and discuss additional targets for imaging which hold great promise for future use in PAH patients.

**Electronic supplementary material:**

The online version of this article (doi:10.1186/2213-0802-1-16) contains supplementary material, which is available to authorized users.

## Introduction

Pulmonary arterial hypertension (PAH) is a deadly disease characterized by vasoconstriction and abnormal remodeling of pulmonary vessels, leading to a progressive increase in pulmonary artery pressure. Key to patient outcome is the response of the right ventricle (RV) to the functional, cellular and molecular alterations in the pulmonary circulation [1–3]. Although rare (PAH has a prevalence of 26 cases per million adult population) [4], the disease occurs most frequently at a young age and is associated with a severely reduced life expectancy [5]. More than ten different drugs are currently registered for the treatment of PAH; all are rather specific pulmonary vasodilators with modest effects on abnormal vascular remodeling. While partial unloading of the heart by these drugs does improve symptoms, there is no cure or major survival benefit after medical treatment [5]. In animal models there is evidence that PAH specific treatments may directly affect RV function, independently from their effects on the pulmonary circulation [6, 7]. The use of cardiac-specific drugs to improve RV function in PAH has only recently been considered and primarily involves interventions in neuro-hormonal signaling and metabolic remodeling [8–10].

The introduction of new compounds for the treatment of PAH and associated RV failure has been seriously hampered by the lack of methods to directly assess the primary disease processes in patients. Neither crude estimates of a subject’s exercise capacity, nor invasively determined hemodynamic profiles provide direct information on the biological responses of the lung vasculature and heart to medical treatments. The distance walked in 6 minutes is used as primary endpoint in most drug trials in PAH, but the validity of this outcome parameter as a surrogate for clinical outcome has been seriously questioned [11]. The fear for complications has prohibited the use of lung or cardiac biopsies to determine organ specific cellular and molecular changes at the time of diagnosis or during follow-up. There is a great unmet need for imaging methods that aid diagnostic classification and which can reflect and predict responses to treatment. In this review, we will discuss the opportunities and limitations of Positron Emission Tomography (PET) to assess lung vascular remodeling and RV adaptation in PAH patients. PET is a non-invasive imaging modality that allows for quantitative *in vivo* measurements of molecular processes. PET imaging has only recently been applied in PAH patients and has far from reached its full potential.

### *In vivo* assessment of lung vascular remodeling

The increased pulmonary vascular resistance in PAH is thought to come about through a combination of vasoconstriction, vascular occlusions and vascular loss. Among the multiple factors implicated in the etiology of these lung vascular abnormalities are genetic mutations, infection and inflammation, increased shear stress and a derailed regulation of processes of cellular growth and apoptosis [12]. Several of the cellular and molecular changes which take place in the PAH vasculature, are potential imaging targets. So far, PET imaging in PAH has been restricted to the quantification of increased glucose uptake and phosphorylation by using 18-fluorodeoxyglucose (^18^F-FDG) tracers. Technical issues specifically pertaining to PET scanning of the lungs are 1) the necessity of corrections for lung density and in-field respiratory movements, and 2) the appropriate choice of input functions. Input functions reflect the supply of radiotracer from the blood to the tissues and are vital for the accurate quantification of PET data. In most applications of PET scanning, tracer activity is measured in the systemic arterial blood to reflect tracer supply to the organs or tissue of interest. If tracers are metabolized during the scanning session, the input function is corrected for the presence of radioactive metabolites and plasma activity concentrations. Identifying an input function for PET imaging of the lungs is complicated due to the existence of dual perfusion systems: the pulmonary and the systemic circulation. This issue is further challenged in PAH by the fact that the relative contributions of the two circulations to total lung perfusion may vary between patients.

#### Aerobic glycolysis: the Warburg effect

A distinct feature of vascular remodeling in animal models and in human PAH is abnormal cellular metabolism, where several cell types in the lung vascular wall, despite ample supply of oxygen, show greater reliance on cytoplasmic glycolysis as opposed to mitochondrial oxidation of glucose or fatty acids [13, 14]. Aerobic glycolysis, or the Warburg effect, is a feature found in malignancies and in non-malignant tissues harboring fast-proliferating cells, including inflammatory cells. ^18^F-FDG PET scanning has been successfully used to quantify increased uptake and phosphorylation of glucose in the PAH lung [13–15], but negative data have also been reported [16]. While discrepancies between studies may be explained by differences in patient preparation, scanning protocols and data analysis (*e.g.* absolute quantification with dynamic PET scanning is likely superior to determining standardized uptake values in a static scan), it is becoming clear that ^18^F-FDG uptake in the PAH lung is highly variable, does not correlate to disease severity or survival, and rapidly normalizes upon PAH treatment [14, 16]. Therefore, clinical applicability of ^18^F-FDG PET scanning in PAH seems limited. For a summary of the advantages and disadvantages of ^18^F-FDG PET scanning in PAH: see Table 1.

**Table 1:** **Clinical advantages and disadvantages of** ^**18**^ **F-FDG PET scanning in PAH**

**Advantages**	• Commonly available in many hospitals
	• Easy to manufacture and easy to transport
	• Mechanisms which predict an altered tracer activity in PAH have been relatively well studied
**Disadvantages**	• No consensus exists on appropriate patient preparation prior to imaging (*e.g.* overnight fasting and glucose loading), leading to inconsistencies in results between centers
	• Lung and right ventricular ^18^F-FDG uptake does not correlate with disease severity or survival
	• Lung and right ventricular ^18^F-FDG uptake rapidly normalize upon PAH treatment, questioning the utility of ^18^F-FDG PET in the follow-up of patients
	• Corrections for lung density have not been performed
	• Increased FDG uptake in the heart could either reflect an increase in
	• After load or a direct, after load-independent, change in energy metabolism. It is not possible to determine the relative contributions of both processes

**Figure 1:**
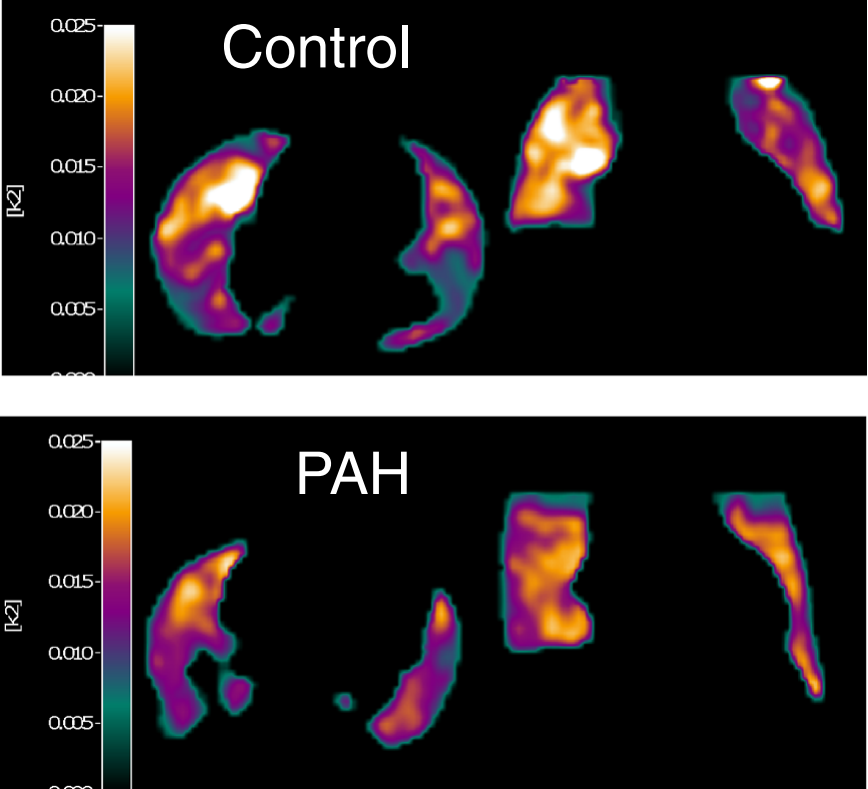
^**11**^ **C-Acetate wash-out scans of the lungs of a control subject (upper panel) and a patient with pulmonary arterial hypertension (PAH, lower panel.** There is a suggestion of a decreased acetate wash-out in the PAH patient, which is compatible with a lower oxygen uptake. This would comply with a metabolic shift in the lung characterized by a decreased glucose oxidation and an increased reliance on anaerobic metabolism. These findings need to be confirmed in a larger group of patients and controls.

In addition to ^18^F-FDG, ^11^C-acetate [17] may have a role as a tracer for PET-imaging based quantification of the Warburg effect in PAH. After its uptake in tissue, ^11^C-acetate is oxidized in the citric acid cycle and its metabolite, ^11^C-CO_2_, is excreted from the tissue [18]. The rate of excretion of tracer activity is a quantitative measure of this oxidation step and, consequently, of oxygen use. When metabolism shifts away from fatty acid oxidation and moves towards glycolysis, as is the case in the PAH lung, ^11^C-acetate excretion rates are expected to be reduced relative to healthy lungs (Figure 1). Measured excretion rates are insensitive to differences in lung density, as lung density only affects the absolute scaling of tracer activity but not tracer clearance rates. In addition, a simplified measure of the excretion rate: K-mono, can be derived by fitting an exponential function through the downslope of the tissue curve [19]. The use of K-mono requires no input function, overcoming some of the technical issues mentioned before. For these reasons, ^11^C-acetate may potentially be used as an alternative to ^18^F-FDG for research and treatment response purposes. However, data confirming this hypothesis are currently lacking and more studies are warranted.

#### Proliferation

Several cell types in the PAH lung exhibit increased proliferation rates, including endothelial cells, smooth muscle cells and adventitial cells (fibroblasts and others) [12]. Proliferation rates are readily picked up by continuous incorporation of thymidine in rapidly dividing cells, which provides another opportunity for imaging of the PAH lung. When ^18^F-labeled fluorothymidine (FLT) is injected into the blood, it builds up in rapidly dividing cells [20] and can be imaged by a PET scanner. This relatively straightforward imaging method has yet to be tested in PAH. Preclinical data shows that the increased growth potential of cells in the PAH lung is also reflected by expression of growth factor receptors, including platelet derived growth factor receptor (PDGF-R) [21], fibroblast growth factor receptor (FGF-R) [22] and epidermal growth factor receptor (EGF-R) [23]. Signaling through these receptors is mediated by receptor tyrosine kinases, and receptor tyrosine kinase inhibitors are not only being developed as drugs, but also as PET tracers [24]. These tracers can perhaps be used to quantify lung vascular remodeling and predict treatment responses in PAH, but this hypothesis remains untested.

#### Extracellular matrix remodeling and angiogenesis

Vascular remodeling in PAH has been compared to a process of uncontrolled angiogenesis [12]. Several steps in the angiogenic process, such as endothelial cell migration, proliferation, survival and differentiation are regulated by integrins. The α_v_β_3_ and α_5_β_1_ integrins are cell adhesion receptors which are over-expressed in the PAH lung in comparison to the normal lung [22, 25] and bind to a variety of extracellular matrix proteins containing an arginine-glycine-aspartic acid (RGD) sequence, such as vitronectin and fibronectin. As such, radiolabeled RGD analogues can perhaps be used for imaging of angiogenesis in PAH. Dijkgraaf *et al.* showed that dimeric RGD peptides accumulate in α_v_β_3_integrin expressing tumors more efficiently than the original monomeric analogues [26] and they developed a dimeric ^18^F galacto-RGD analogue: (cRGDfK)2-DOTA for PET imaging [27]. A tracer currently under development is a matrix metalloproteinase (MMP)-9 tracer, which would allow assessment of extracellular matrix remodeling. Other tracers which could be of specific interest to study vascular remodeling in PAH are *e.g.* markers of transforming growth factor β and bone morphogenic protein signaling [28].

### *In vivo* imaging of right heart adaptation and failure

The response of the RV to the increased load in PAH is the end-result of multiple interactions between autocrine, paracrine and neuro-endocrine signaling pathways, together determining contractility, cardiomyocyte hypertrophy and survival, mitochondrial function and metabolism and extracellular matrix composition [29]. Accurate determination of RV adaptation is highly desirable, not only when caring for individual patients but also when conducting clinical trials evaluating new treatments. A major technical limitation of cardiac PET scanning, however, is its low spatial resolution together with the presence of cardiac and respiratory movements. The small size of the normal RV prohibits use of the method in control subjects, and therefore the method has only been used to assess treatment responses in patients, or to compare patients with different disease severities. Cardiac gating of dynamic scans may improve the quality of right ventricular imaging, perhaps allowing accurate assessments of the RV in even control subjects.

#### RV blood flow and oxygen balance

In PAH, pressure overload increases RV work and thereby augments the demand for myocardial blood flow and oxygen supply. Both can be estimated using PET. Using a combination of H_2_^15^O, ^15^O_2_ and C^15^O tracers, it was shown that the increased myocardial O_2_ demand in PAH patients is primarily determined by elevated pulmonary pressures and heart rate [30, 31]. Whereas the normal blood supply to the RV has a considerable reserve in oxygen extraction, the myocardial oxygen extraction fraction (OEF) of resting PAH patients is significantly increased (~70% as opposed to 45-50% in dogs) [30, 32], leaving little opportunity to increase oxygen extraction during exercise. To maintain myocardial oxygen supply during exercise, RV coronary flow would have to increase. This does not seem to occur in PAH patients [30], who may have an impaired right coronary blood flow [33]. PET studies have demonstrated that RV O_2_ supply during exercise is restricted [34]. Using adenosine stress perfusion Cardiac Magnetic Resonance, it was shown that LV and RV perfusion reserves are both decreased in PAH, which suggests that a systemic component plays a role in the development of RV dysfunction [35]. An imbalance in RV O_2_ demand and supply in PAH patients can result in ischemia, as has been illustrated by Gomez *et al.* using stress induced SPECT scintigraphy [36].

#### RV metabolism

Whether or not associated to a decrease in oxygen supply, the RV of PAH patients shows several changes in metabolism culminating in an increased glucose uptake. While the normal heart predominantly uses fatty acids as energy source, the overloaded heart switches to glucose to preserve adenosine triphosphate (ATP) supply [37]. In addition, metabolic remodeling in the pressure overloaded RV results in a greater reliance on anaerobic energy generation in the form of glycolysis [38], similarly as for the lungs. These metabolic changes resemble the changes found in lung vascular cells and have been confirmed by PET scanning in PAH patients, showing decreased fatty acid uptake (using ^11^C-palmitate as surrogate) [39] and increased ^18^F-FDG uptake in the RV. Decreases in pulmonary vascular resistance with vasodilator treatment have been shown to correlate with decreases in ^18^F-FDG uptake [40]. RV ^18^F-FDG uptake is numerically associated with a worse clinical profile and survival [15, 41–44], but in most studies, correlations between ^18^F-FDG uptake and hemodynamic parameters were moderate at best. There was also a considerable discrepancy between the different studies. Recent preclinical evidence suggest that the metabolic shift towards glycolysis is not sustained during the progression of RV failure [45], which would question the use of ^18^F-FDG uptake as a reliable biomarker in PAH.

#### Angiogenesis and extracellular matrix remodeling

Preclinical studies have shown that insufficient angiogenesis in the setting of rapid RV hypertrophy results in ischemia and fibrosis [45, 46]. Vascular endothelial growth factor (VEGF) is the major contributor to angiogenesis and adhesion molecules such as integrins are other important angiogenic modulators. By use of PET imaging with ^64^Cu-labeled VEGF_121_ or ^18^F RGD with affinity for the α_v_β_3_ integrins, it has been possible to measure angiogenic myocardial repair in rat models of myocardial infarction [47–49]. These imaging techniques allow longitudinal monitoring of therapeutic responses and may become important in the study of pathophysiological pathways of myocardial disease processes *in vivo*.

#### Neuro-hormonal signaling

Chronic activation of the sympathetic nervous and renin-angiotensin systems have been demonstrated in PAH patients and experimental models and are important players in progression of the disease in the heart and lungs [9]. Myocardial norepinephrine uptake is quantified by PET using the ^11^C-*meta*-hydroxyephedrine (HED) tracer. Pietilä showed the prognostic significance of a reduced HED uptake in heart failure patients [50], which probably reflects down-regulation of myocardial beta-receptor density due to chronic hyperactivity of the sympathetic nervous system. Post-synaptic β-adrenergic receptor density can also be assessed using the ^11^C-CGP-12177 tracer [51], but neither of these tracers has been used in PAH. The same is true for tracers which allow activity assessment of the renin angiotensin system [52].

### Hybrid imaging: PET-CT or PET-MR

The ideal imaging modality in PAH would combine *in vivo* evaluation of pulmonary and RV function and structure, with detailed spatial information of molecular processes. PET-CT (CT: computed tomography) is readily available in the clinical and research setting and its use has significantly contributed to our insights in pathophysiological processes in cardiovascular disease. Most relevant to imaging in PAH, CT allows accurate determination of lung anatomy and tissue density. CT is less useful than magnetic resonance (MR) imaging when it comes to functional assessments of the RV. PET-MR is an innovative, rapidly emerging technique [53–55] which allows simultaneous anatomic and functional imaging (whereas PET and CT scans are made sequentially), quantification of perfusion and tissue characterization. The major limitation of hybrid systems with MR is that no information is obtained which allows attenuation correction of nuclear images. Although it is particularly challenging to measure density variations in the lungs, this has become an active area of research [56, 57].

### Development of new tracers

Scientific developments lead to a continuous identification of potential treatments and new molecules relevant to the pathobiology of PAH and associated RV failure. Many of these compounds and molecules have the potential to be developed into imaging targets. The development of a new tracer is a costly and labor-intensive process, however. After identification of a promising compound, the novel tracer has to undergo several validation steps before it can be used as a radiotracer in more extensive clinical (research) studies. First, the actual radio-synthesis has to be performed using a synthetic route that is reproducible and has a sufficiently high yield. Next, preclinical studies are needed to establish stability of the compound *in vivo* (*e.g.* ingrowth of radiolabeled metabolites), to assess whether the signal is related to the biological process under study (*e.g.* blocking studies in case of a receptor ligand) and to develop a GMP compliant radio synthesis route. Finally, proof of concept studies need to be performed in humans. These proof of concept studies typically involve test-retest studies in small groups of patients and normal subjects to assess whether uptake is different between both subject groups. These studies are also used to develop a quantitative tracer kinetic model and to determine the variability of the model.

## Review

While the pathobiological understanding of PAH and associated RV failure is increasing, there is still a great need to develop methods which can assess disease severity, and predict and monitor responses to treatment. Imaging of the most pertinent cellular and molecular processes in the lungs and heart by means of PET scanning is still undeveloped, but holds great promise. Increased proliferation rates, altered cellular metabolism and angiogenesis are those disease processes which seem most suitable to assess pulmonary vascular remodeling in PAH. RV adaptation and failure can be assessed by some of the same tracers, and also by quantification of blood flow and oxygen supply. It is unlikely that PET imaging will enter the diagnostic algorithm of PAH, as the currently used tracers do not provide information specific to the pathobiology of PAH or any other condition associated with a high pressure in the lung (*e.g.* chronic thrombo-embolic pulmonary hypertension). Moreover, there is no data estimating the possible sensitivity of PET imaging in PAH. However, PET scanning may have a role in the quantification of the disease processes underlying PAH and PAH associated RV failure. As such, PET imaging may be of great interest for the purpose of treatment response monitoring. Because the development of new tracers is costly and labor intensive, further development of PET scanning in a rare disease such as PAH requires a multicenter, multinational effort geared towards rapid identification of new targets and efficient development of new tracers.

## Conclusions

PET provides the opportunity to image and quantify relevant disease processes in PAH and associated RV failure, including proliferation, angiogenesis, matrix remodeling, shifts in metabolism and neurohormonal signaling. The main role of PET imaging in PAH patient management lies in the monitoring of treatment responses. With the development of additional imaging targets, PET holds a great promise for future use in PAH patients.

## Authors’ original submitted files for images

Below are the links to the authors’ original submitted files for images. Authors’ original file for figure 1

## Abbreviations

CT: Computed tomography
EGF-R: Epidermal growth factor receptor
FGF-R: Fibroblast growth factor receptor
18F: 18-fluoro
FDG: Fluoro-deoxyglucose
FLT: Fluorothymidine
MMP-9: Matrix metalloproteinase 9
MR: Magnetic resonance
PDGF-R: Platelet derived growth factor receptor
PAH: Pulmonary arterial hypertension
PET: Positron emission tomography
RGD: Arginine-glycine-aspartic acid
RV: Right ventricle
