# Supplementary figures and images for: Pulmonary vascular remodeling and right heart failure in pulmonary hypertension: future role of positron emission tomography in decoding the enigma
Source: Transl Respir Med. 2013 Dec 11;1:16. doi: 10.1186/2213-0802-1-16 (PMC4715170; doi:10.1186/2213-0802-1-16)

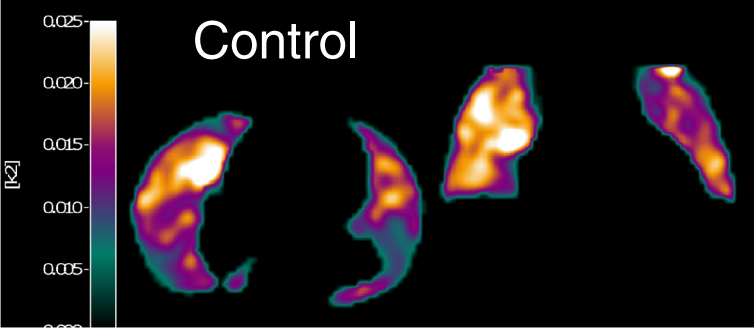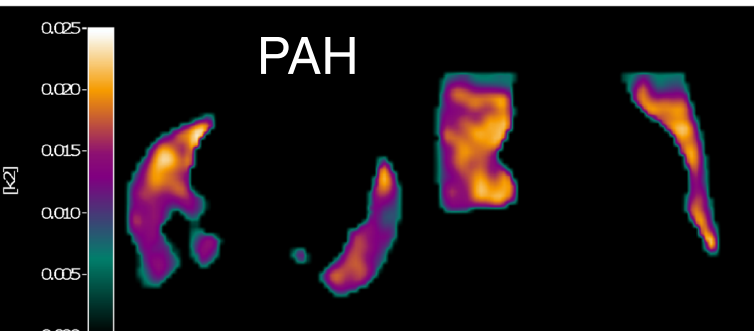

Supplement: Supplementary file 1 — Authors’ original file for figure 1 [file 40247_2013_23_MOESM1_ESM.pdf]
